# Novel *NOTCH3* alteration expanding the molecular spectrum of pericytic tumours: report of two cases

**DOI:** 10.1111/his.70051

**Published:** 2025-11-20

**Authors:** Irena Antonia Ungureanu, Megane Le Quang, Rihab Azmani, Marie Ancelle, Henri Margot, Guillaume Chotard, François Le Loarer, Nathalène Truffaux

**Affiliations:** ^1^ Department of Pathology Paris‐Saclay University, Versailles SQY University (UVSQ), Ambroise‐Paré Hospital Boulogne‐Billancourt France; ^2^ Department of Pathology University Hospital of Bordeaux, Pellegrin Hospital Bordeaux France; ^3^ Bioinformatics, Data and Digital Health Department Bergonié Institute, Comprehensive Cancer Center Bordeaux France; ^4^ Medical Genetics Department University Hospital of Bordeaux Bordeaux France; ^5^ Department of Pathology University of Bordeaux, INSERM U1218 ACTION, Bergonié Institute Bordeaux France; ^6^ Department of Pathology Bergonié Institute Bordeaux France

**Keywords:** internal tandem duplication, molecular, myofibromas, NOTCH3, pericytic

## Abstract

**Introduction:**

Myofibromas are part of the pericytic tumour family, which includes myopericytomas, glomus tumours and angioleiomyomas. While they typically display benign behaviour when arising in the skin and subcutaneous tissues of the head and neck, rare aggressive variants have been reported, particularly those with visceral or intracranial involvement. The most frequently identified molecular alterations in myofibromas are *PDGFRB* gain‐of‐function mutations, primarily single‐nucleotide substitutions.

**Case presentation:**

Herein, we report two cases of myofibroma: one aggressive case with central nervous system involvement in a newborn, exhibiting a monophasic morphology, and a second, subcutaneous case in an adult. RNA sequencing was performed on both tumours, and data analysis was conducted using a four‐pipeline fusion‐calling approach (Arriba, FusionCatcher, FusionMap and STAR‐Fusion). This analysis identified a novel somatic internal tandem duplication (ITD) in the *NOTCH3* gene, affecting exons 26 and 27, specifically involving the C‐terminal heterodimerization domain of the NOTCH3 receptor. Unsupervised hierarchical clustering demonstrated that myofibromas with ITDs segregate distinctly from conventional myofibromas and other pericytic tumours.

**Discussion and conclusion:**

Our findings suggest that *NOTCH3* ITDs represent a novel oncogenic mechanism in pericytic tumour pathogenesis, likely driving constitutive activation of the NOTCH signalling pathway. Given the potential therapeutic relevance, particularly in aggressive or life‐threatening cases with CNS involvement, our findings highlight the importance of extensive molecular profiling. Targeted therapy with NOTCH inhibitors may represent a promising strategy in the management of aggressive cases of ITD‐driven pericytic tumours.

**Abbreviations**
FFPE: formalin‐fixed, paraffin‐embedded
GSI: Gamma‐secretase inhibitor
HD: heterodimerization domain
ITD: internal tandem duplication
NRR: negative regulatory region

## Introduction

Myofibromas are benign pericytic tumours that share a similar pericytic lineage with myopericytomas, glomus tumours and angioleiomyomas. They predominantly occur in infants (sometimes referred to as infantile myofibroma, IM) and young children, typically arising in the skin and subcutis of the head and neck, where they generally exhibit benign behaviour. In some cases, they manifest in visceral or intracranial sites, showing an aggressive progression associated with a high mortality if left untreated. This aggressive behaviour can also occur in the setting of synchronous multiple lesions referred to as myofibromatosis, which may happen in sporadic or familial cases and typically affects infants and young children.^1^, ^2^, ^3^

At the molecular level, various driving alterations have been identified in these tumours. Myofibromas and myofibromatosis are commonly associated with *PDGFRB* gain‐of‐function mutations in more than 70% of cases, most of which are single‐nucleotide substitutions or small insertion–deletion events. Rare *SRF* rearrangements have been evidenced in a cellular variant of myofibroma. Myopericytomas can also be associated with *PDGFRB* mutations and, in some cases, with *NOTCH2* rearrangements.^4^, ^5^, ^6^, ^7^, ^8^

Myofibromatosis may threaten the life of patients when occurring in high‐risk locations such as the skull base due to its rapid growth and invasion potential. Therefore, the identification of all potential targetable alterations is important to improve the management of these threatening cases. The use of tyrosine kinase inhibitors has indeed shown significant efficacy in the management of familial infantile myofibromatosis with *PDGFRB* alterations.^9^, ^10^, ^11^ In this article, we report novel mutations involving *NOTCH3* in two cases of pericytic tumours, detected by total RNA sequencing. We emphasize the potential therapeutic significance of this novel alteration.

## Cases Presentation

### Case no. 1

**Figure 1:**
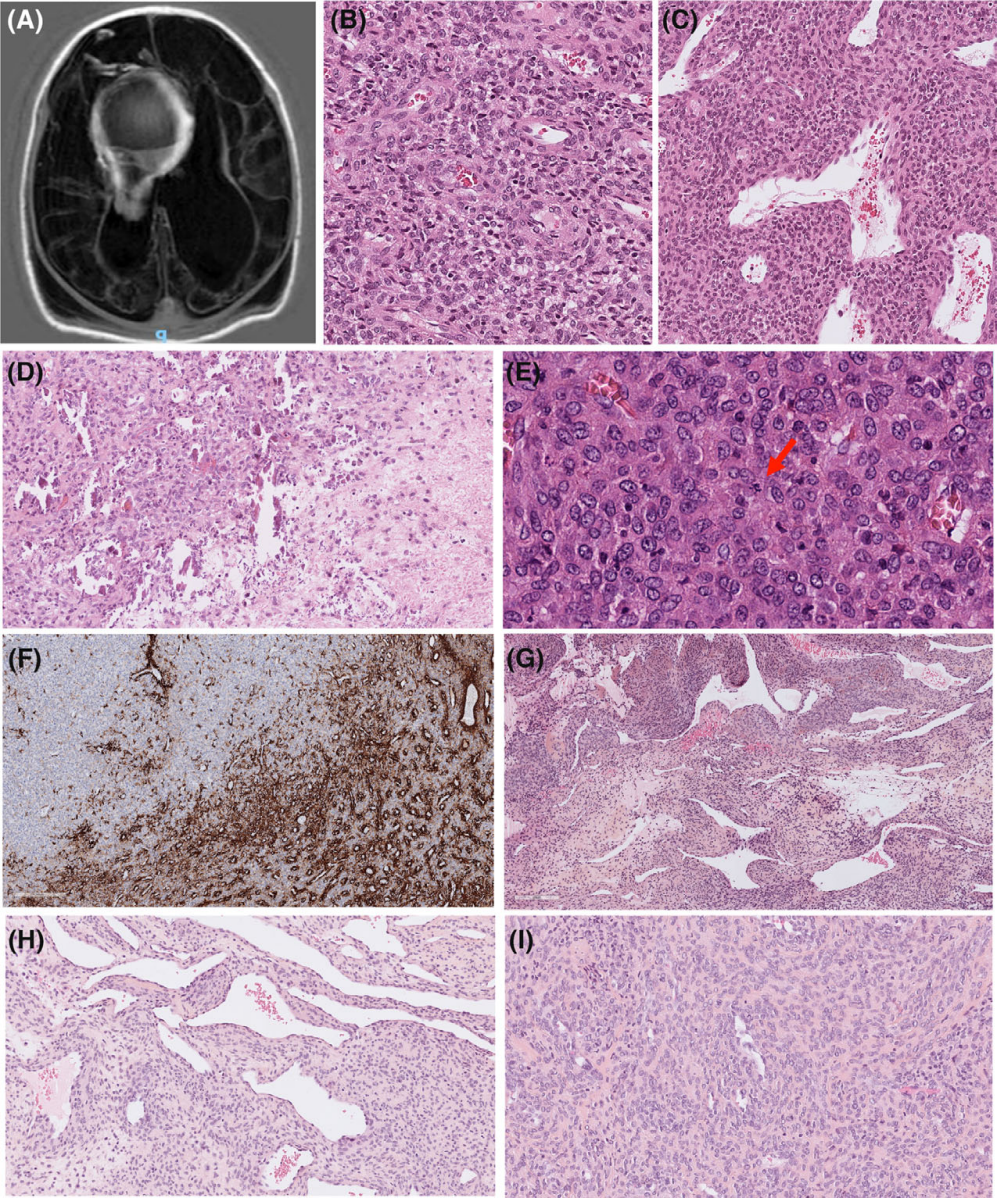
Histological and radiological features of *NOTCH3*‐mutated myofibromas. Case 1. Myofibroma in a newborn located in the central nervous system (**A–F**). (**A**) Axial T1‐weighted inversion recovery (IR) magnetic resolution imaging reveals a large mass located in the third ventricle, characterized by solid, cystic, and calcified areas, associated with biventricular hydrocephalus. (**B**) Histology reveals neoplastic cells with a perivascular arrangement (HES, 200×) (**C**) Tumour cells are arranged in solid sheets with hemangiopericytic vessels, lacking stroma and merging with the vascular walls (HES, 100×). (**D**) Areas of coagulative necrosis (at the right) and foci of calcifications are observed within the lesion (HES, 35×) (**E**) Neoplastic cells display oval nuclei with clear chromatin and well‐defined nuclear membranes. Atypical mitoses were present (arrow) (HES, 400×). (**F**) Neoplastic cells show partial positivity for smooth muscle actin (immunohistochemistry, 100×). Case 2. Subcutaneous myofibroma in a 43‐year‐old man (**G–I**). (**G**) At low‐power magnification, the tumour proliferation harbours striking variation of cellularity and prominent hemangiopericytic vessels with ovoid cells arranged in perivascular sheaths, highlighting pseudopapillary formations due to protrusions into the vascular lumina (HES, 40×). (**H**) This region shows that tumour cells are intermingled with a rich hemangiopericytic vascular network with onion rings‐like arrangements around the capillaries (HES, 30×). (**I**) The neoplastic cells display bland, monotonous oval nuclei with inconspicuous cytoplasm showing an angiocentric distribution surrounding small capillaries (HES, 45×).

The first case involved a male newborn, delivered at term, who presented at birth with severe axial hypotonia, poor temperature regulation, no visual tracking and abnormal movements. Magnetic resolution imaging revealed a large well‐circumscribed cerebral mass, measuring 56 × 36 × 35 mm, centred on the third ventricle, with solid, cystic and calcified components associated with intracystic haemorrhage. Secondary multicystic encephalomalacia affecting both cerebral hemispheres was associated, as a complication resulting from the mass (Figure 1A). The infant's condition rapidly worsened, leading to death a few days later. A post‐mortem biopsy was performed and sent for histopathological examination. Microscopic examination revealed a highly cellular neoplastic proliferation associated with extensive areas of coagulative necrosis and foci of calcifications. The tumour cells had an immature round to epithelioid cytomorphology and were arranged in solid sheets and short fascicles. Tumour cells were centred on vessels and showed budding around and within well‐developed hemangiopericytic vessels. The nuclei were monotonous with clear chromatin and well‐defined nuclear membranes. The cells displayed an eosinophilic cytoplasm without distinct borders. Tumour necrosis was absent. Mitotic activity was noted, including atypical figures, with 11 mitoses/mm^2^. Immunohistochemical analysis revealed partial positivity with smooth muscle actin, and no reactivity for CD34, desmin and ICN1 in tumour cells (Figure 1B–F). The proliferation index ki67 was around 40%. RNA was extracted from formalin‐fixed, paraffin‐embedded (FFPE) block tissue. Total RNA sequencing was performed using multiple four fusion calling pipeline (Arriba, FusionCatcher, FusionMap and STAR‐Fusion). An internal tandem duplication (ITD) of exon 26 L1614_V1629 of the *NOTCH3* gene (NM_000435.3) was found. The mutation was not found in the germline upon genetic analysis performed at the parents' request. A final diagnosis of IM was established with morphological features fitting with a monophasic cellular form.

### Case no. 2

A 43‐year‐old man presented with a 6 cm subcutaneous mass in the scalp. The lesion was excised and sent for histopathological examination. The sample consisted of multiple fragments of a neoplastic proliferation made of ovoid to spindle‐shaped cells forming intersecting fascicles embedded within a dense fibrous stroma. The vascular network was prominent, focally haemangiopericytic, and neoplastic cells crowded around the vessels or protruded into the vascular lumina. The nuclei of the tumour cells were uniform in shape, ovoid or spindled, with clear chromatin and a fine nucleolus along with inconspicuous cytoplasm (Figure 1G–I). No mitosis was seen and the KI‐67 proliferation index amounted to 5%. A diagnosis of myofibroma was established based on morphological features. The patient was lost to follow‐up and no clinical outcome data were available.

The initial RNA sequencing analysis on the FFPE block tissue did not reveal any fusion or mutation. The reads were specifically reanalysed using the same bioinformatic tools described in the previous case, showing an ITD of exon 27 L1644_P1645ins16 of *NOTCH3* according to Arriba.

**Figure 2:**
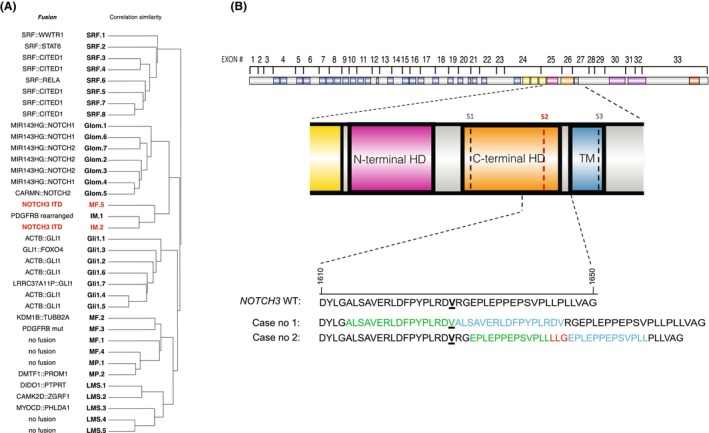
(**A**) Unsupervised hierarchical clustering of pericytic tumours including the two index cases IM.2 and MF5 (in red) (SRF: *SRF*‐rearranged pericytic tumour, Glom: glomus tumour, MF: myofibroma, IM: infantile myofibroma, Gli: *GLI1*‐rearranged tumour, MP: myopericytoma, LMS: leiomyosarcoma). (**B**) Schematic representation of the protein structure and predicted NOTCH3 protein sequences from both cases. The bottom part shows the wild‐type NOTCH3 protein sequence (amino acids 1610–1650) and the predicted mutant sequences from each case. The first and second segments of the duplicated sequences are depicted in green and blue, respectively. In case no. 2, the ITD adds three novel junctional amino acids (in red). (N‐terminal HD: N‐terminal heterodimerization domain, C‐terminal HD: C‐terminal heterodimerization domain, TM: transmembrane domain, S1‐3: proteolytic cleavage sites). The dotted lines indicate cleavage sites S1‐3.

The unsupervised hierarchical clustering analysis based on RNA expression profiles showed that this case clustered tightly with the other index case, compared with conventional myofibromas (*n* = 4), myopericytomas (*n* = 2) and glomus tumours (*n* = 7). Both cases clustered tightly (MF.5 and IM.2, Figure 2A) together with a case of myofibromatosis associated with the canonical *PDGFRB* alteration (Figure 2).

## Discussion

We report herein a novel type of mutation in myofibromas, involving the *NOTCH3* gene, which expands the molecular spectrum of these tumours. Alterations involving *NOTCH* genes are well known in pericytic tumours. *NOTCH2* is fused in 5′ to *MIR143* and *CARMN* partners in glomus tumours, while *NOTCH3* mutations involving exon 25 have been linked to angioleiomyomas, myopericytomas and glomus tumours.^5^, ^12^ Of note, one study has previously reported a single case of familial infantile myofibromatosis associated with a *NOTCH3* germline alteration in the form of a missense substitution (c.4556T>C, Leu1519Pro), affecting the conserved heterodimerization domain (HD) of the protein.^13^

In case 1, the tumour displayed a predominantly monophasic growth pattern composed of immature cells, deviating from the classical biphasic morphology of myofibroma but akin to the immature monophasic variant seen in infantile myofibromatosis.^14^ Classically, myofibromas exhibit alternating fascicles of spindle‐shaped cells with eosinophilic cytoplasm and elongated nuclei, with areas with a hemangiopericytoma‐like vascular pattern populated by more primitive round cells.

The monophasic/immature variant is challenging to diagnose and may be mistaken for a malignant paediatric sarcoma such as a rhabdomyosarcoma or the recently identified *SRF*‐rearranged myoid neoplasms, sometimes also referred to as cellular myofibroma as they share overlapping morphologic features with pericytic tumours but harbour commonly a smooth muscle phenotype.^15^, ^16^ Additionally, the differential may also include a cellular meningioma, which tumorigenesis is interestingly driven by NOTCH3 signalling.^17^

In this study, we describe new ITDs, a genetic alteration previously described in *FLT3* in acute myeloid leukaemia and in a subset of *BCOR*‐rearranged sarcomas that may harbour *BCOR* ITDs.^18^, ^19^ This type of alteration is notoriously difficult to identify from short‐read RNA sequencing due to the low sensitivity of usual alignment tools for mutation detection. These medium‐sized duplications require appropriate bioinformatic software such as Arriba which has shown high performance in detecting ITDs and should be implemented in bioinformatics pipeline analysis of whole RNA sequencing in routine analysis.^20^

These ITDs are localized in exons 26 and 27 of *NOTCH3* that encode for the C‐terminal HD, containing two key proteolytic cleavage sites—S1 for receptor maturation and S2 for its activation (Figure 2B). In the Notch signalling pathway, the ligand binding generates a mechanical force that unfolds the negative regulatory region (NRR) and exposes the S2 site on the Notch receptor.^21^ This exposure permits subsequent proteolytic cleavage by an ADAM‐family metalloprotease, followed by gamma‐secretase cleavage at a third site (S3), leading to Notch intracellular domain release and nuclear signalling.^21^ Both identified ITDs closely flank the key proteolytic site S2 and are predicted to modify its conformation by duplicating 17 or 16 amino acids in the C‐terminal HD. These duplications may disrupt interdomain interaction with the NRR, potentially leading to ligand‐independent constitutional activation of Notch signalling. These alterations could therefore be eligible for anti‐NOTCH therapy in aggressive cases of IM/myofibromatosis such as the one described here. Further data are needed to confirm this hypothesis.

While NOTCH inhibitors are currently under development, Gamma‐secretase inhibitors (GSIs), monoclonal antibodies and non‐coding RNA therapies have shown clinical potential in treating malignant glomus tumours and other cancers.^21^, ^22^ For instance, a paediatric case of metastatic glomus tumour harbouring a *CARMN::NOTCH1* fusion had a significant response to LY3039478, a GSI that blocks the release of the Notch intracellular domain.^23^ Other anti‐NOTCH drugs such as pan‐Notch inhibitors and antibody‐drug conjugates have shown promising results in clinical studies.^24^, ^25^

Overall, pericytic tumours seem to share deregulation of PDGFRB and NOTCH signalling, which are key pathways for vascular development and homeostasis. PDGF ligands are encoded by four different genes (*PDGFA‐D*), and two genes encode PDGF receptors (PDGFR‐α and ‐β). Notch signalling is important for the development of both endothelial and mural cells, dictating arterial and venous fates of angioblasts. Some studies showed that PDGFR‐β expression is upregulated by Notch ligand induction or by activated forms of the Notch receptor. PDGFRB expression has been shown to be upregulated by activated Notch signalling.^26^ Additionally, mutations such as NOTCH3^L1519P^ have been demonstrated to increase PDGFRB expression, showing the relationship between the two pathways, where Notch signalling predominates over PDGF pathways.^27^ Interestingly, the cases of myofibroma identified in our total RNA sequencing results (Figure 2A) formed two distinct clusters. The main cluster included most of the myofibroma cases (*n* = 4), among them one with *PDGFRB* mutation and two myopericytomas. The smaller cluster (*n* = 3) comprised the two index cases and a *PDGFRB‐*rearranged myofibromatosis. This latter cluster segregated closer to the clade of *NOTCH*‐altered glomus tumours, suggesting similar deregulation of NOTCH signalling in this subset of tumours characterized by NOTCH ITDs.

In conclusion, our study reports a novel *NOTCH3* ITD alteration found in both infantile and adult myofibromas that could lead to NOTCH3 signalling activation. ITDs should be systematically screened during molecular analysis and adequate tools are required to detect them. Our study is also a reminder that IM involving the CNS may be life threatening. Neuropathologists should be familiar with these mesenchymal tumours. Precise knowledge of their underlying molecular drivers may help clinicians manage these rare dramatic cases with adjuvant medical therapy.

## Author contributions

IAU, NT and FLL wrote the main manuscript. IAU, GC and MLQ prepared Figure 1. NT, RA and MA performed molecular analysis and prepared Figure 2. HM performed genetic analysis. GC and MLQ provided clinical data.

## Conflict of interests

The authors declare that they have no conflict of interest.

## Consent

The histopathological reviews were conducted in the Netsarc+ healthcare network with written information on the procedure for health and research analysis. According to French law (Loi Jardet), the absence of opposition from the patient enables the publication of research conducted without new medical procedures (research setting MR004).

## Data Availability

The datasets used and/or analysed during the current study are available from the corresponding author on reasonable request.
